# Carnosine supplementation and retinal oxidative parameters in a high-calorie diet rat model

**DOI:** 10.1186/s12886-023-03255-y

**Published:** 2023-12-08

**Authors:** Rogil Jose de Almeida Torres, Fernando Moreto, Andrea Luchini, Rogerio Joao de Almeida Torres, Sofia Pimentel Longo, Ricardo Aurino Pinho, Seigo Nagashima, Lucia de Noronha, Artur Junio Togneri Ferron, Carol Cristina Vagula de Almeida Silva, Camila Renata Correa, Giancarlo Aldini, Ana Lucia Anjos Ferreira

**Affiliations:** 1 Medical School, Department of Internal Medicine, Universidade Estadual Paulista (UNESP), Botucatu, SP 18618-687 Brazil; 2 Department of Ophthalmology, Centro Oftalmologico de Curitiba, Curitiba, PR Brazil; 3 Department of Ophthalmology, Hospital Angelina Caron, Campina Grande Do Sul, PR Brazil; 4 Postgraduate Program in Health Sciences, School of Medicine, Pontificia Universidade Catolica Do Paraná, Curitiba, PR Brazil; 5 Dipartimento Di Scienze Farmaceutiche (DISFARM), Università Degli Studi Di Milano, Milan, Italy

**Keywords:** High-calorie diet, Carnosine, Retina, Antioxidant

## Abstract

**Background:**

To assess oxidative effects induced by a high-calorie diet on the retina of Wistar rats and test the antioxidative effects of carnosine supplementation.

**Methods:**

Wistar rats were randomly divided into the following groups: standard diet (SD), high-calorie diet (HcD), standard diet + carnosine (SD + Car), and high-calorie diet + carnosine (HcD + Car). The body weight, adiposity index, plasma glucose, total lipids, high-density lipoprotein (HDL), low-density lipoprotein (LDL), uric acid, creatinine, and triglycerides of the animals were evaluated. The retinas were analyzed for markers of oxidative stress. Hydrogen peroxide production was assessed by 2',7'-dichlorodihydrofluorescein diacetate (DCF) oxidation. The total glutathione (tGSH), total antioxidant capacity (TAC), protein carbonyl, and sulfhydryl groups of the antioxidant system were analyzed.

**Results:**

TAC levels increased in the retinas of the SD + Car group compared to the SD group (*p* < 0.05) and in the HcD + Car group compared to the HcD group (*p* < 0.05). The levels of GSH and the GSSH:GSSG ratio were increased in the HcD + Car group compared to the SD + Car group (*p* < 0.05). An increase in the retinal carbonyl content was observed in the HcD group compared to the SD group (*p* < 0.05) and in the HcD + Car group compared to the SD + Car group (*p* < 0.05). A high-calorie diet (HcD) was also associated with a decrease in retinal sulfhydryl-type levels compared to the SD group (*p* < 0.05).

**Conclusion:**

The results suggest that feeding a high-calorie diet to rats can promote an increase in carbonyl content and a reduction in sulfhydryl groups in their retinas. The administration of carnosine was not effective in attenuating these oxidative markers.

**Trial registration:**

Animal Ethics Committee of Botucatu Medical School - Certificate number 1292/2019.

## Background

Consumption of a high-calorie diet without an appropriate amount of simple carbohydrates causes metabolic disorders in the body, which result in morphofunctional alterations in various tissues that are directly associated with the aging process [1, 2]. This type of diet induces excessive production of reactive oxygen species (ROS), mainly superoxide anions, through the mitochondrial electron transport chain and is associated with obesity, hypertension, hyperglycemia, hyperinsulinemia, and dyslipidemia [3–5]. Redox imbalance damages the structural integrity of biomolecules, influencing the mechanisms of tissue repair [6–8]. In this scenario, redox imbalance and the inflammatory process, the main pathogenic pillars of diseases induced by metabolic syndrome, are also closely related to age-related macular degeneration (AMD) pathogenesis [9, 10]. Age-related macular degeneration is the main cause of blindness in elderly individuals. A systematic review and meta-analysis revealed that 8.7% of the world’s population has developed AMD, and the number of AMD-affected people is projected to reach 288 million in 2040 [11]. Despite the large investments in experimental and clinical research, the best results have only enabled vision care providers to delay central vision loss. Hence, it is important to acknowledge and act on modifiable factors by eliminating or attenuating the factors, with the objective of slowing the onset of this disease as much as possible.

Carnosine (L-carnosine) is a cytoplasmic dipeptide (β-alanil-L-histidina) found in high concentrations (millimolar) in the skeletal muscle, liver, intestine and brain of vertebrates and invertebrates [12–14]. Carnosine may be obtained through a diet; however, it is not absorbed in its whole form, as the carnosinase enzyme present in the digestive system rapidly hydrolyzes the compound [15–18]. This natural product exhibits antioxidative properties, eliminating ROS in the cells [19–23] and reducing the lipid hydroperoxides produced by the oxidation of polyunsaturated fatty acids (PUFAs) in cellular membranes [24, 25]. Other effects include its ability to sequester unsaturated aldehydes and stimulate muscle glucose uptake [26, 27]. These biological roles are of extreme importance for obese individuals affected by insulin resistance and/or metabolic syndrome [28, 29]. Several studies have addressed the properties of carnosine as an antioxidant, immunomodulator and neuroprotective agent [15]. The beneficial effects of carnosine are attributed to its anti-advanced glycation end product (AGE) and -advanced lipoxidation end product (ALE), as well as to its anti-inflammatory properties [30]. However, the molecular mechanisms that explain these effects have not been clearly defined. Studies indicate that carnosine acts by a direct antioxidant mechanism and by sequestering reactive carbonyls (RCS), the byproducts of lipid and glucose oxidation; thus, AGE and ALE, which are the reaction products of RCS with proteins, are inhibited [30]. Moreover, carnosine has been found to act indirectly by activating the transcription factor nuclear factor e2-related factor 2 (Nrf2), a mechanism that explains many of the effects evoked by this peptide, such as anti-inflammatory, antioxidant, antiglycation and anti-carbonyl effects that lead to its therapeutic power [31, 32]. In the ocular globe, high levels of L-carnosine (approximately 25 µM) were found in human transparent crystalline lenses, whereas the level of ripe human cataracts decreased, reaching 5 µM [33]. Studies with bioactive N-acetylcarnosine (NAC) ophthalmic prodrug lubricant eye drops revealed their efficacy in preventing and treating cataracts [34, 35]. Carnosine, through the inhibition of nuclear factor-κB (NF-κB) expression and oxidative stress, also plays a significant role in the survival of mouse retinal ganglion cells after optic nerve crush [36]. Additionally, oral carnosine treatment protected retinal capillary cells after 6 months of experimental hyperglycemia. This protection was not caused by the prevention of ROS or AGEs but was associated with a significant induction of Hsp27 in activated glial cells and normalization of increased Ang-2 levels in diabetic retinas [37]. Carcinine, another peptide that resembles carnosine, is a natural antioxidant with hydroxyl-radical-scavenging activity that promotes the neuroprotection of photoreceptor cells after exposure to strong light [38]. To our knowledge, no studies have addressed the effect of carnosine on the retinas of obese subjects.

Considering that diet and obesity are modifiable factors of AMD [39], a hypothesis was formulated in the current study that a high-calorie diet can induce oxidative alterations in the retina of rats. Simultaneously, the antioxidative effects of carnosine supplementation on the retinas of these animals were tested.

## Methods

### Animals and experimental protocol

All experiments and protocols were approved by the Animal Ethics Committee of Botucatu Medical School (1292/2019) and were performed in accordance with the Use of Animals in Ophthalmic and Vision Research and the National Institute of Health Guidelines for the Care and Use of Laboratory Animals and ARRIVE guidelines. Male Wistar rats (189 ± 9 g, 8 weeks of age) were housed in an environmentally controlled room (22 °C ± 3 °C; 12 h light–dark cycle and relative humidity of 60 ± 5%). After the tenth week, the animals were randomly distributed into two groups. The group with 19 animals received a normocaloric diet (SD), and the group with 15 animals was fed a high-calorie diet (HcD) rich in simple carbohydrates for 20 weeks. In the twentieth week, the two groups (SD and HcD) were subdivided into two new groups. One of the SD groups (SD, *n* = 9) continued to receive the normocaloric diet, whereas the other SD group was fed the normocaloric diet and carnosine intraperitoneally (250 mg/kilogram) (SD + Car, *n* = 10). One of the HcD groups continued to receive the high-calorie diet (HcD, *n* = 7), whereas the other was fed the high-calorie diet and infusions of carnosine (250 mg/kilogram) (HcD + Car, *n* = 8). The whole experiment lasted 24 weeks, which included 20 weeks of induction and 4 weeks of treatment. The HcD groups also received water + sucrose (25%). The diets and water were provided ad libitum.

### Administration of carnosine

Carnosine (L-carnosine, CarnoPure™, Flamma Group, Chignolo d’Isola, Italy) was administered intraperitoneally (250 mg/kilogram) for 4 weeks (5 consecutive days) after the induction period [40]. Infusion was performed with a 30 G needle, and the injected volume was approximately 500 µL.

### Diets

The normocaloric diet included a formulation of adequate macro- and micronutrients for rats [41]. The high-calorie diet also respected micronutrient adequacy, although the main source of energy was derived from simple carbohydrates (75 to 80% of the total calorific value of the diet) supplemented by the marginally increased percentage of lipids. To reach the increased percentage values of simple carbohydrates, 25% sucrose solution was also added. Both diets were prepared at the UNIPEX Diet Center at the School of Medicine of Botucatu. The consumption of water and food by all animals was measured weekly. The diets used in this study were designed in our laboratory.

The HcD diet contained soybean meal, sorghum, soybean peel, dextrin, sucrose, fructose, lard, vitamins, and minerals, plus 25% sucrose in drinking water. The normocaloric diet contained soybean meal, sorghum, soybean peel, dextrin, soy oil, vitamins, and minerals. The nutrients and nutritional composition of each diet are presented in Table 1.

**Table 1:** Diet composition and nutritional values

Components	SD	HcD
Soybean meal (g/kg)	335	340
Sorghum (g/kg)	278	80
Soy hulls (g/kg)	188	116
Dextrin (g/kg)	146	20
Sucrose (g/kg)	——	80
Fructose (g/kg)	——	180
Soybean oil (g/kg)	14	——
Lard (g/kg)	——	154
Minerals (g/kg)	25	25
Salt (g/kg)	4	8
Nutritional values		
Protein (% of ingredients)	20.0	18.0
Carbohydrate (% of ingredients)	60.0	53.5
Fat (% of ingredients)	4.00	16.5
% of unsaturated	69.0	47.0
% of saturated	31.0	53.0
% Energy from protein	22.9	16.6
% Energy from carbohydrate %	66.8	49.2
Energy from fat	10.4	34.2
Energy (kcal/g)	3.59	4.35

*SD* Standard diet, *HcD* high sugar-fat diet

### Nutritional analysis

The nutritional profile was evaluated according to the following parameters: food and caloric intake and body weight. Food consumption was measured daily, and body weight was measured weekly. Caloric intake was determined by multiplying the energy value of each diet (g × Kcal) by the daily food consumption. For the HcD group, caloric intake also included calories from water (0.25 × 4 × mL consumed).

### Metabolic analysis

Blood was collected from the tail after 12 h of fasting and just prior to sacrifice, and plasma was used to measure biochemical parameters. Glucose concentration was determined by using a glucometer (Accu-Chek Performa; Roche Diagnostics, Indianapolis, IN, USA); triglycerides and uric acid were measured with an automatic enzymatic analyzer system (Chemistry Analyzer BS-200, Mindray Medical International Limited, Shenzhen, CN). The levels of basal glucose, total lipids, triglycerides, high-density lipoprotein (HDL), and low-density lipoprotein (LDL) were determined by using colorimetric techniques (BT-330 spectrophotometer, Biosystems).

### Obesity characterization

Body weight was measured weekly to establish the presence of obesity, which was based on weight gain and the adiposity index. The weight gain was calculated by subtracting the initial weight from the final weight of the animals [weight gain (g) = final weight (g) − initial weight (g)]. The adiposity index represents the ratio of the sum of the epididymal, visceral, and retroperitoneal fat deposits by the final weight multiplied by 100 [adiposity index (%) = (epididymal (g) + visceral (g) + retroperitoneal (g))/final weight (g) × 100].

### Sample preparation

After the 24th week, the rats were sacrificed via decapitation under deep isoflurane anesthesia, and the eyes were slid open with a sharp surgical knife. With the aid of a dissecting microscope, the neural retina layer was carefully peeled off from the posterior section of the eye and placed on weighing paper. The retinas were then scraped out, placed in a plastic vial, homogenized with 800 µL of phosphate-buffered saline (PBS) and stored in a -80 °C freezer for further oxidative stress analysis.

### Oxidative stress assays

#### ROS production

Hydrogen peroxide production was assessed by oxidation of 2’,7'-dichlorodihydrofluorescein diacetate (DCF) and measured by flow cytometry. The samples were homogenized in 1:1 (v/v) PBS at room temperature. Then, 20 µL of the sample was incubated in 50 µL of DCF (10 µM) at 37 °C for 30 min, and the formation was stopped at 4 °C. The formation of the fluorescent derivative of oxidized DCF was monitored with excitation and emission wavelengths of 488 and 525 nm, respectively, using a BD Accuri C6 Cytometer.

#### Antioxidant system

The total glutathione (tGSH) levels were assessed based on a reaction between GSH and 5,5-dithio-bis(2-nitrobenzoic acid) (DTNB; Ellman’s Reagent, Sigma Aldrich Corporation, St. Louis, MO, USA), which generated an oxidized glutathione-TNB product that was later reduced by glutathione reductase in the presence of nicotinamide adenine dinucleotide phosphate (NADPH), consequently generating GSH. The oxidized GSH (GSSG) was measured using the recycling of GSSG through monitoring NADPH in the presence of 2-vinylpyridine with spectrophotometric techniques. The GSH and GSSG concentrations were determined using a regression curve from various GSH or GSSG standards [42]. According to the manufacturer’s instructions, the antioxidant equivalent concentrations were measured at 570 nm as a function of Trolox concentration. Total antioxidant capacity (TAC) was measured using a colorimetric assay kit (Sigma Aldrich Corporation, St. Louis, MO, USA). The antioxidant equivalent concentrations were measured at 570 nm as a function of the Trolox concentration described above. Sulfhydryl groups were measured as described previously [43]. Samples were diluted at a 1:6 ratio in 0.1 M sodium phosphate containing 1 mM EDTA (pH 8.0), and 100 μL of this dilution was reacted with 50 μL (4 mg/mL) of DTNB. After an incubation period of 15 min at room temperature, sample absorbance was measured at 412 nm using a microplate reader (Versamax, Molecular Devices, USA). Concentrations of sulfhydryl groups were determined by parallel measurements of an L-cysteine standard curve. Protein carbonylation was determined by the reaction of 2,4-dinitrophenylhydrazine (DNPH) with carbonyl, generating an adduct absorbed at 366 nm [44]. Oxidative stress was also expressed by the GSH/GSSG ratio.

### Statistical analysis

The results are displayed as the mean ± standard error of the mean (SEM). Differences among groups were determined by one-way analysis of variance (ANOVA) followed by the Newman‒Keuls post hoc test when appropriate. Differences with *p* < 0.05 were considered statistically significant. All statistical analyses were performed using GraphPad Prism 8 software (version 8).

## Results

### Body weight, adiposity index, and serum metabolic parameters

The initial average body weight was 189 ± 9 g, ensuring sample homogeneity at the beginning of the experiment. At the end of the experiment, HcD animals displayed a higher body weight than the SD + Car group (*p* < 0.05). Statistical significance was not observed among the other groups (Table 2). The highest adiposity index was observed in the groups fed the hypercaloric diet (*p* < 0.05), revealing no carnosine effect (Table 2). Glucose plasma levels were higher in the HcD and HcD + Car groups than in the SD group (*p* < 0.05). No significant differences were observed among the other groups (Table 2). Our results also showed that the HcD e HcD + Car groups presented significantly increased plasma triglyceride levels compared to the SD and SD + Car groups (*p* < 0.05), whereas the HcD + Car group contained significantly higher serum triglyceride levels than the SD and SD + Car groups (*p* < 0.05) (Table 2).

**Table 2:** Nutritional and plasma- metabolic parameters

Variable	**Group**			
	SD (*n* = 9)	HcD (*n* = 7)	SD + Car (*n* = 10)	HcD + Car (*n* = 8)
Body weight (g)	503.5 ± 17.0	561.3 ± 15.4	490.4 ± 11.1^a^	566.0 ± 32.5
Adiposity index (%)	4.15 ± 1.6^a, b^	8.51 ± 1.0	3.11 ± 1.1^a, b^	7.67 ± 1.6
Glucose (mg/dL)	79.2 ± 2.3^a, b^	87.6 ± 2.6	88.8 ± 3.0	93.1 ± 3.2
Triglycerides (mg/dL)	81.3 ± 7.6^a, b^	171.7 ± 25.2	51.3 ± 4.9^a, b^	137.9 ± 19.3^a^

Data expressed as means ± standard error of mean (SEM); groups

One-way ANOVA followed by Newman-Keuls post-hoc test (*p* < 0.05) was used to compare groups

*SD* standard diet, *SD* + *Car* standard diet + carnosine, *HcD* high sugar-fat diet, *HcD* + *Car* high sugar-fat diet + carnosine

^a^different from HcD

^b^different from HcD + Car

### Levels of 2',7'-dichlorodihydrofluorescein Diacetate (DCF)

**Fig. 1:**
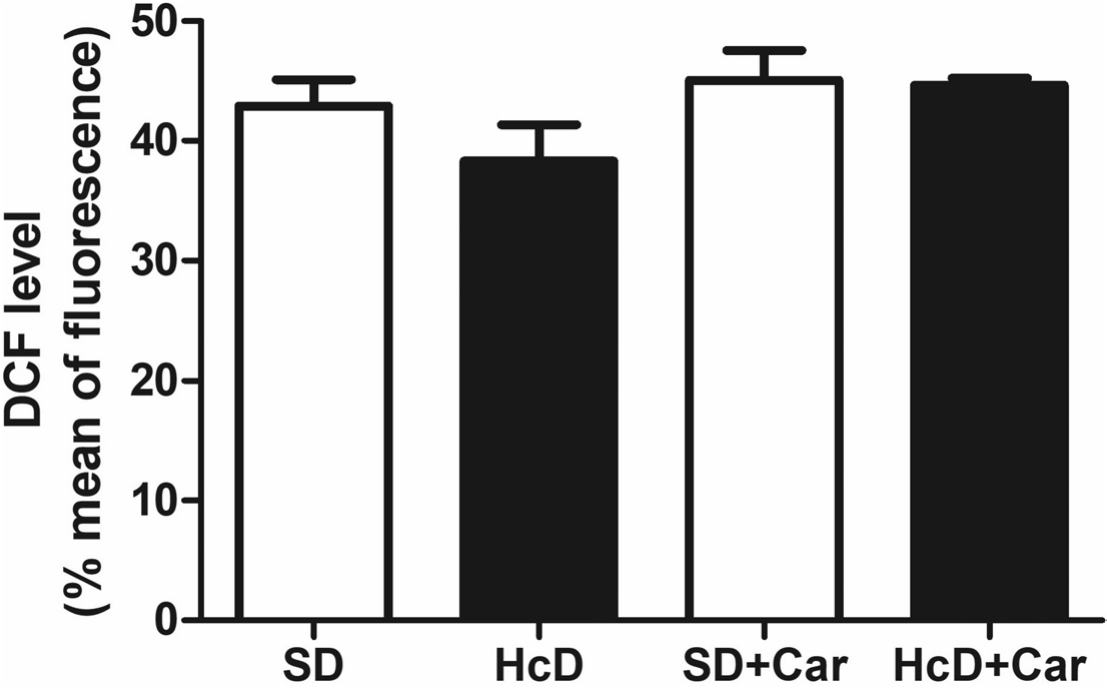
Carnosine supplementation effects on hydrogen peroxide production in animals fed a high caloric diet. Values are means + \- standard error of the mean (SEM); groups: SD, standard diet; SD + Car, standard diet + carnosine; HcD, high-calorie diet; HcD + Car, high-calorie diet + carnosine. Carnosine 250 mg / (Kg body wt /day) or saline IP for 4 wks; DCF, formation of the fluorescent derivative of oxidized DCFH-DA (oxidation of 2 ', 7'-dichlorodihydrofluorescein diacetate); One-way ANOVA, followed by the Newman-Keuls test, used to analyze the treatment effects

The production of oxidants is shown in Fig. 1 (DCF levels). No differences were observed among the groups; administration of diet and carnosine produced no effect.

### Antioxidant system in the retina

**Fig. 2:**
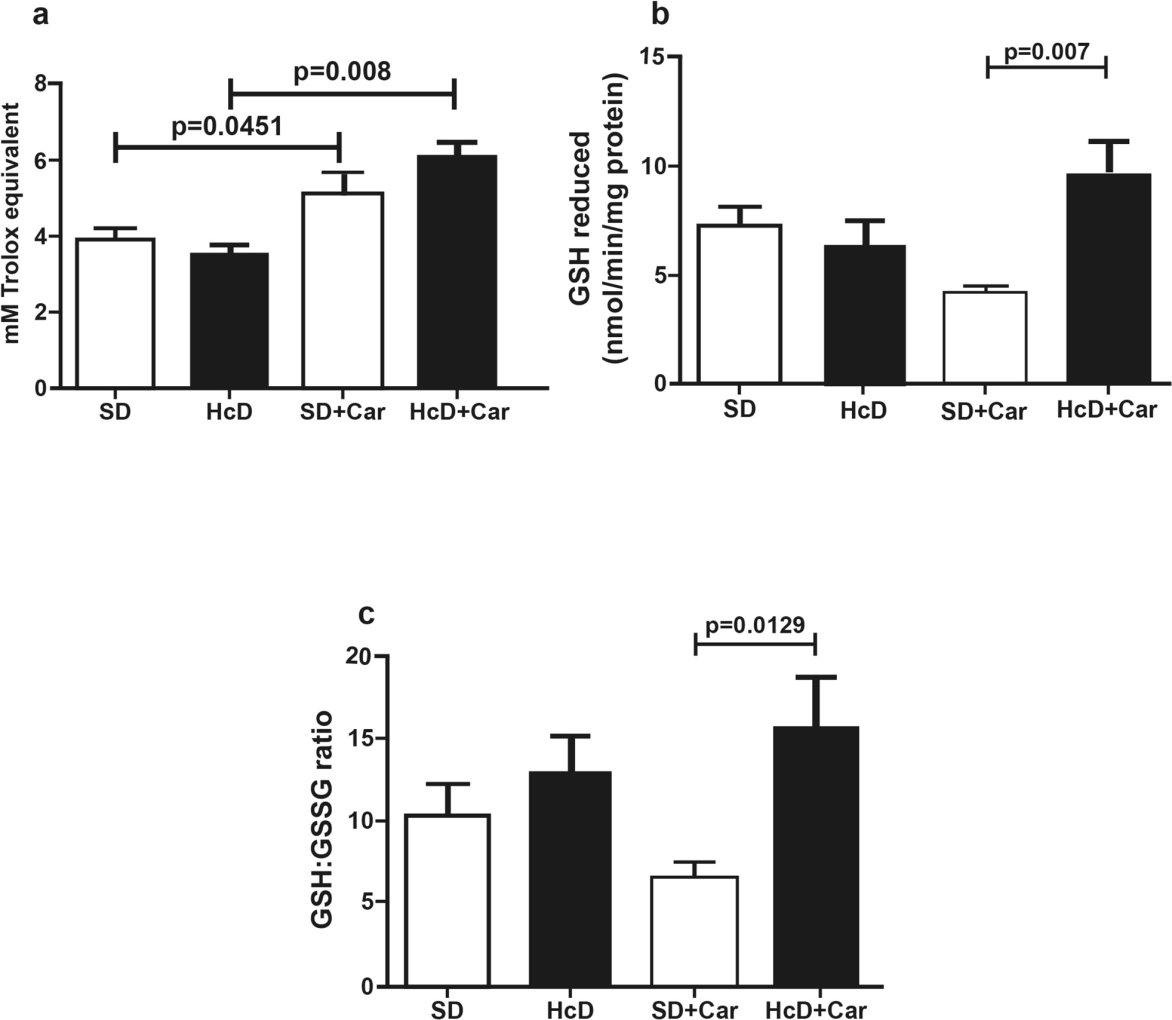
Effect of carnosine supplementation on antioxidant system in retinas from animals undergoing a high-calorie diet. Values are means + \- standard error of the mean (SEM); groups: SD, standard diet; SD + Car, standard diet + carnosine; HcD, high-calorie diet; HcD + Car, high high-calorie diet + carnosine. Carnosine 250 mg / (Kg body wt /day) or saline IP for 4 wks; Total antioxidant capacity (TAC) (**a**), total GSH activity (**b**), and glutathione (GSH)/oxidized glutathione (GSSG) ratio (**c**). One-way ANOVA, followed by the Newman-Keuls test, used to analyze the treatment effects (*p* < 0.05)

The absence of a diet (HcD) effect was verified in retina TAC levels. However, carnosine supplementation increased TAC levels in the SD + Car group compared to the control (SD) group (*p* < 0.05) and the HcD + Car group compared to the HcD group (*p* < 0.05) (Fig. 2a). The diet (HcD) did not influence retina-reduced GSH levels. Carnosine supplementation increased GSH levels in the HcD + Car group compared to the SD + Car group (*p* < 0.05) (Fig. 2b). The GSH:GSSG ratio was not affected by the diet. Carnosine supplementation increased the GSH:GSSG ratio in the HcD + Car group compared to the SD + Car group (*p* < 0.05) (Fig. 2c).

**Fig. 3:**
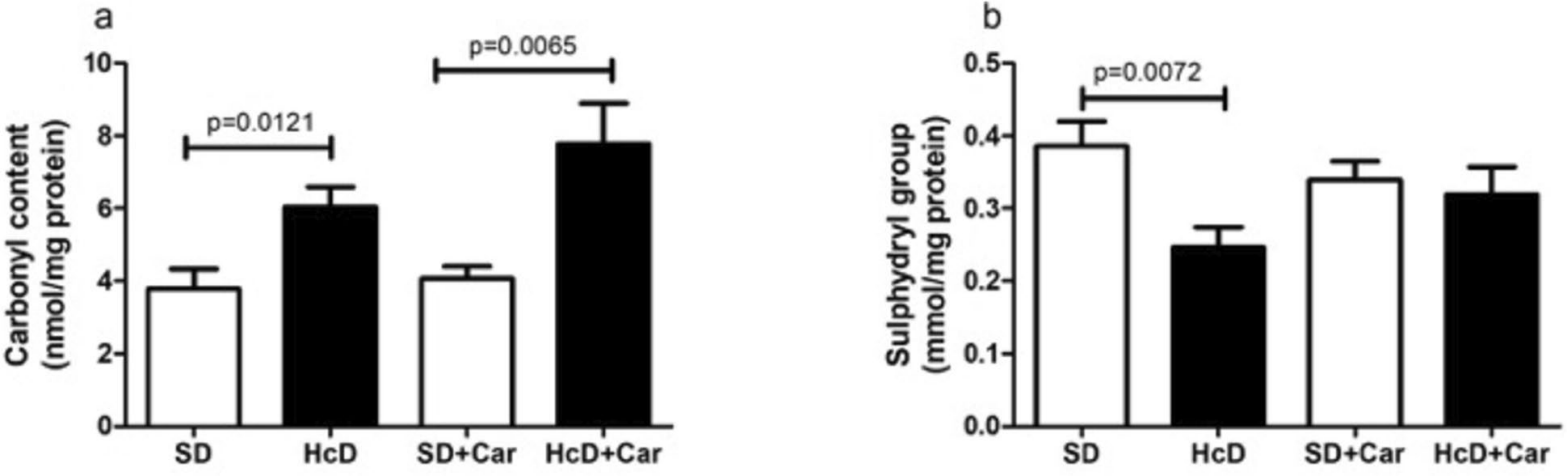
Effects of carnosine supplementation on carbonyl contents and sulfhydryl levels in the retina. Values are means + \- standard error of the mean (SEM); groups: SD, standard diet; SD + Car, standard diet + carnosine; HcD, high-calorie diet; HcD + Car, high-calorie diet + carnosine. Carnosine 250 mg / (Kg body wt /day) or saline IP for 4 wks; Carbonyl contents, **a**, sulfhydryl type levels, **b**; One-way ANOVA, followed by the Newman-Keuls test, used to analyze the treatment effects (*p* < 0.05)

The high-calorie diet was associated with an increase in retinal carbonyl content compared to the normal diet (HcD > SD) (*p* < 0.05). Carnosine supplementation was related to parameter enhancement in carnosine-supplemented rats treated with a high-calorie diet compared to the SD + Car group (HcD + Car > SD + Car) (*p* < 0.05) (Fig. 3a). Diet (HcD) was also associated with a decrease in retinal sulfhydryl-type levels (HcD < SD) (*p* < 0.05). The absence of a carnosine supplementation effect was verified in this parameter (Fig. 3b).

## Discussion

**Fig. 4:**
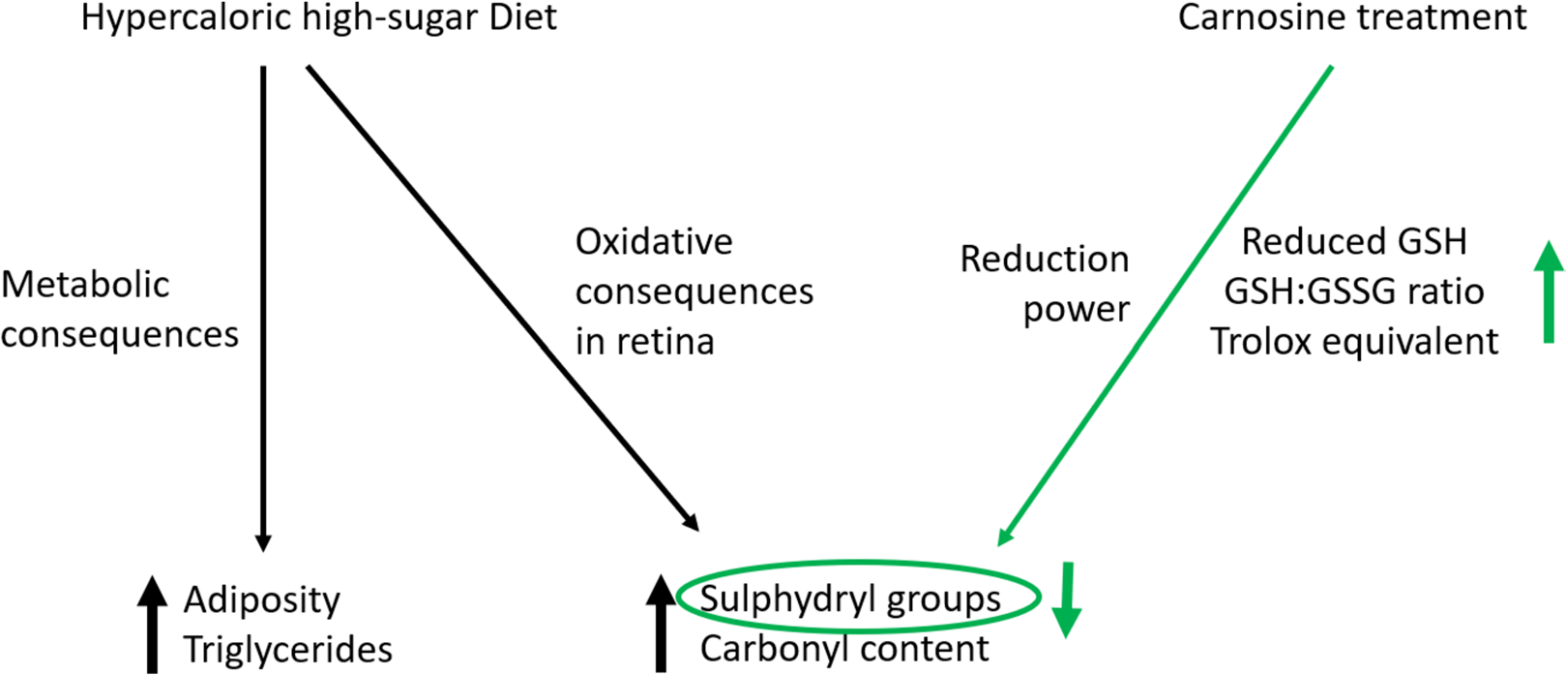
Effects of high-calorie diet and carnosine supplementation on metabolic parameters and retinal oxidation

The aim of this study was to assess the oxidative effects induced by a high-calorie diet on the retina of Wistar rats and test the antioxidative effects of carnosine supplementation. The experimental model proposed in this study promoted metabolic changes, which are represented by increased levels of fasting blood glucose and plasma triglycerides. Carnosine supplementation influenced plasma triglycerides, although without statistical significance. Greater weight gain and a different body composition represented by the adiposity index were also observed. In retinal tissue, the high-calorie diet did not influence the redox state. Compared to the SD and HcD groups, the groups that received carnosine (SD + Car and HcD + Car) exhibited an increase in TAC. The high-calorie diet group that received carnosine (HcD + Car) also presented higher GSH concentrations and a higher GSH:GSSG ratio than the SD + Car group. Oxidative damage associated with the high-calorie diet, characterized by a higher concentration of carbonylated proteins and lower concentrations of sulfhydryl groups, was observed. However, treatment with carnosine only induced a nonsignificant increase in sulfhydryl-type levels. Figure 4 summarizes these findings.

Simple carbohydrates and saturated fatty acids are associated with the development of metabolic syndrome and multiple comorbidities [45–47]. In relation to vision, the Beaver Dam Eye Study associated high intake of saturated fat and cholesterol with an increased risk for early AMD [48]. In addition, compared to foods with a low glycemic index (fruits, cereals, vegetables, whole wheat bread), foods with high glycemic indices contributed 2.71 times more to the development of AMD, which corroborated the results obtained in this study [49]. The present study used a high-calorie diet enriched with simple carbohydrates with 25% sucrose solution. This innovative model mimics the development of obesity with metabolic complications, according to a protocol previously adopted by our group [41]. This high-calorie diet can induce hyperadiposity, insulin resistance with hyperglycemia, dyslipidemia, and even arterial hypertension, as Wistar rats hardly develop hypertension via diet models [41]. In this model, echocardiographic and renal function alterations were observed [41]. For clarification purposes, it is important to note that “control” animals (SD) are usually fed ad libitum, which frequently leads to overeating and excessive gain of body weight [50, 51]. This knowledge was confirmed at the end of the study when weight gain was observed for the animals in the four groups in relation to the start of the experiment. The amount of energy obtained by the animals fed ad libitum significantly exceeded their energy expenditure, resulting in a substantial gain of body weight or positive energy balance, which is frequently associated with early diseases [52]. Consequently, the group that ingested food ad libitum may have been subjected to harmful factors, which were induced by an intake above body requirements [53]. For this reason, our study comprised the following groups: the SD group; HcD group; SD + Car group; and HcD + Car group. As expected, the high-calorie diet induced a significant increase in glucose in relation to the SD group, corroborating literature findings [54]. However, the addition of carnosine (HcD + Car) could not reverse this condition. The high-calorie diet also induced a significant increase in serum triglyceride (TG) levels. Similar results were attained in studies on diets high in refined sugar [3, 55]. Carnosine supplementation led to a decrease in triglycerides in relation to HcD, although with no statistical significance.

The high-calorie diet group received carnosine supplementation with the objective of testing the antioxidant effects of carnosine on the retina. High-calorie diet intake, associated with a Western lifestyle, has been correlated with excessive generation of ROS [3–5]. It is known that a sucrose-based diet interferes with the performance of the antioxidant system, overloading the organism’s defense system and leading to oxidative stress [56–58]. In addition, studies have demonstrated that high fructose and high-fat diets affect the function and structure of the rat retina [59]. In our study, the high-calorie diet did not interfere with the production of hydrogen peroxide in the retina, as shown in Fig. 1, with no significant difference among groups.

The levels of total antioxidant activity (TAC), expressed in mM Trolox equivalents, were higher in the retinas of the groups that received carnosine supplementation than in the retinas of the groups that did not receive carnosine (Fig. 2a). Notably, a low TAC value directly indicates the deficit of its specific composing substances [60], and TAC an important biomarker of the tissue antioxidant system [61]. The TAC levels in plasma were lower in patients with AMD compared to a control group [60, 62–66]. This likely indicates that the oxidoreduction disturbance may be involved in the pathogenesis of AMD and that the increase in TAC levels may be associated with a protective factor of the retina. In this regard, carnosine may have played a protective role in the retinas of the rats fed the high-calorie diet.

The high-calorie diet group (HcD) presented a decrease in reduced GSH levels in relation to the SD group, but this decrease was not statistically relevant. It has been observed that a high sucrose diet (545 g/kilogram of sucrose) administered for more than three months induced a significant decrease in GSH in the rat brain [58]. Most likely, the smaller amount of sucrose (80 g/kilogram) offered to HcD animals in the present study may account for the discreet reduction in GSH, corroborating the findings of another study [67]. The contribution of glutathione deficiency to oxidative stress was an important finding, indicating that glutathione deficiency may play a key role in the pathogenesis of many diseases [68]. Conversely, the HcD + Car group showed a significant increase in GSH levels in relation to the SD + Car group (Fig. 2b). Importantly, glutathione performs several major physiological functions, such as protection of cells against destructive effects of reactive oxygen intermediates and free radicals, detoxication of external substances such as drugs and environmental pollutants, maintenance of red cell membrane stability, and enhancement in immunological function through its effects on lymphocytes [69]. It was shown that oxidation-induced apoptosis of RPE cells may be protected by GSH [70]. Another study reported significantly lower plasma GSH in older individuals affected by AMD, diabetes, and controls (elderly with no diabetes or AMD) than in younger individuals [71]. Considering the increase in GSH in the HcD + Car group, carnosine may play a protective role in the retina.

A decrease in the GSH:GSSG ratio was observed in the HcD group compared with the HcD + Car group, although with no significant differences. Aging, chronic diseases, a high-calorie diet, and AMD may reduce the GSH:GSSG ratio [71–74]. As known, the activity of GSH as an antioxidant can be expressed as a function of GSH concentration and as a function of the redox state of the GSH:GSSG ratio [71]. In the present study, the high-calorie diet did not influence the retinal redox state. However, treatment with carnosine in HcD animals promoted an increase in GSH concentrations and the GSH:GSSG ratio in relation to SD animals. The high-calorie diet did not cause changes in the tested oxidative markers (TAC, GSH, and GSH:GSSG ratio), preventing further analysis of the antioxidative effects of carnosine in the adopted model.

The present research also mapped the protein carbonylation of the Wistar rat retina fed hypercaloric diets, as high values of protein carbonyl groups have been found in patients with AMD [62, 75]. Reactive carbonyl species are important cytotoxic mediators produced due to the oxidative damage caused by biomolecules (lipids and sugars), which alters the cell signaling mechanisms to the nucleus, positively regulates redox-sensitive transcription factors, and induces irreversible structural modification in important molecules [proteins, peptides (cysteine, lysine, histidine), lipids, DNA] [76]. Protein carbonyls are the most widely studied markers of protein oxidation and are frequently used as markers of oxidative stress, as they indicate the amount of protein that has been oxidized by highly reactive free radicals [77, 78]. Due to this ROS overproduction, increased protein carbonylation levels have been described along with these diet-induced disorders [79, 80]. Our results show that long-term intake of a high-calorie diet was associated with the formation of carbonyl functional groups in relation to SD groups (Fig. 3a). As previously demonstrated, a high-calorie diet generates similar results in the plasma and liver [75]. Studies have revealed that carnosine acts by a direct antioxidant mechanism and by sequestering reactive carbonyls (RCS), the byproducts of lipid and glucose oxidation; thus, AGE and ALE, which are the reaction products of RCS with proteins, are inhibited [30]. The in vitro capacity of carnosine to scavenge acrolein and form a 3-methylpyridinium carnosine adduct has been demonstrated [8, 27]; however, in the present study, the dietary intervention of rats supplemented with carnosine did not show specificity by downregulating carbonylation in the retina. On the other hand, an increase in carbonyl protein levels was observed in the HcD + Carn group. This increase did not indicate that carnosine induced higher levels of carbonyl in HcD, as there was no increase in the level of SD + Car in relation to SD.

In addition to the oxidative damage to proteins that a high-calorie diet causes, it was observed that this diet leads to a significant loss of the sulfhydryl group (Fig. 3b). Supplementation of carnosine in the high-calorie diet group restored sulfhydryl levels in the retina to levels similar to those in the SD group (Fig. 3b). This is possibly due to the increased GSH concentrations [81]. Importantly, sulfhydryl groups (also biomarkers of oxidative stress) are the most powerful and most frequent antioxidants found in the plasma [82], and their expression is reduced in AMD patients [83].

A controversial point of this study was that the HcD group did not present retinal oxidation when measured by DCF. It is known that a high-calorie diet causes oxidative stress by increasing the levels of oxidation products in an experimental model [84]. DCF oxidation is widely used for the total detection of ROS, including hydroxyl radicals (•OH) and nitrogen dioxide (•NO2) [85]. Therefore, the high-calorie diet was expected to induce a significant increase in the production of reactive oxygen species measured by DCF. However, the high-calorie diet, as well as the administration of carnosine, did not change this marker. This controversial result was already observed in another experiment [86]. Furthermore, a study that aimed to evaluate the prevention of retinal vascular damage by treatment with oral carnosine after 6 months of experimental hyperglycemia also showed no significant changes in markers of oxidative stress [37]. For this reason, in the future, other ROS detection techniques could be explored to confirm the oxidative status of retinas in the HCD group. Researchers should consider using dihydroethidium on paraffin retinal slides and measuring any ROS source in retinal homogenates (e.g., NADPH oxidase or mitochondria) or the level of retinal nitrosylation.

The diet prepared for this study, which mimics modern eating habits, may trigger an imbalance of some markers of the retina redox state. In this regard, this study strengthens the knowledge that this diet should be reduced or avoided. Carnosine was not effective against the oxidative parameters of HcD groups, such as the carbonyl content and sulfhydryl group. However, carnosine elevated TAC and GSH concentrations in SD retinal tissue. Although some studies found that carnosine helps prevent and cure cataracts, the action of this antioxidant was not tested in oxidative and inflammatory markers of AMD. More studies are needed to determine possible antioxidative and anti-inflammatory effects on AMD and other eye diseases that are potentially associated with metabolic syndromes, such as diabetic retinopathy, hypertensive retinopathy or even glaucoma.

## Conclusions

The high-calorie diet did not influence the tested oxidative markers, such as TAC, GSH, and the GSH:GSSG ratio, but was associated with a significant increase in protein carbonylation and a decrease in sulfhydryl type levels in the animal retina, suggesting that oxidant activity occurs in the retina. The administration of carnosine was not effective in attenuating these oxidative markers.

## Data Availability

The data underlying this article were provided by Ricardo A Pinho with permission. Data will be shared upon request to the corresponding author with permission from Ricardo A Pinho.

## Abbreviations

AGE: Anti-advanced glycation end product
ALE: Advanced lipoxidation end product
AMD: Age-related macular degeneration
DCF: Dichlorodihydrofluorescein diacetate
DNPH: 2,4-Dinitrophenylhydrazine
GSSG: Oxidized GSH
HcD: High-calorie diet
HDL: High-density lipoprotein
LDL: Low-density lipoprotein
NAC: N-acetylcarnosine
NADPH: Nicotinamide adenine dinucleotide phosphate
NF-κB: Nuclear factor-κB
Nrf2: Nuclear factor e2-related factor 2
PBS: Phosphate-buffered saline
RCS: Reactive carbonyls
ROS: Reactive oxygen species
TAC: Total antioxidant capacity
tGSH: Total glutathione
